# Microsurgical Reconstruction of Extensive Oncological Scalp Defects

**DOI:** 10.3389/fsurg.2015.00044

**Published:** 2015-09-30

**Authors:** Ole Goertz, Leon von der Lohe, Ramón Martinez-Olivera, Adrien Daigeler, Kamran Harati, Tobias Hirsch, Marcus Lehnhardt, Jonas Kolbenschlag

**Affiliations:** ^1^Department of Plastic Surgery, BG University Hospital Bergmannsheil, Ruhr-University, Bochum, Germany; ^2^Neurosurgical Unit, Department of Surgery, BG University Hospital Bergmannsheil, Ruhr-University, Bochum, Germany

**Keywords:** plastic surgery, reconstruction, oncology, calvarial defect, head

## Abstract

Although most small to medium defects of the scalp can be covered by local flaps, large defects or complicating factors, such as a history of radiotherapy, often require a microsurgical reconstruction. Several factors need to be considered in such procedures. A sufficient preoperative planning is based on adequate imaging of the malignancy and a multi-disciplinary concept. Several flaps are available for such reconstructions, of which the latissimus dorsi and anterior-lateral thigh flaps are the most commonly used ones. In very large defects, combined flaps, such as a parascapular/latissimus dorsi flaps, can be highly useful or necessary. The most commonly used recipient vessels for microsurgical scalp reconstructions are the superficial temporal vessels, but various other feasible choices exist. If the concomitant veins are not sufficient, the jugular veins represent a safe back-up alternative but require a vessel interposition or long pedicle. Post-operative care and patient positioning can be difficult in these patients but can be facilitated by various devices. Overall, microsurgical reconstruction of large scalp defects is a feasible undertaking if the mentioned key factors are taken into account.

## Introduction

The scalp covers the calvarium and consists of skin, subcutaneous tissue, the galea aponeurotica, loose areolar tissue, and the pericranium. If this cover is disrupted by trauma or the resection of malignancies, the exposed bone can succumb to infection and its potentially life-threatening complications. Therefore, timely reconstruction of such defects is paramount.

For small and medium sized defects, local flaps are often sufficient. Such coverage by means of adjacent tissue gives a good color and texture match and even allows the reconstruction of the hair bearing area. However, in large defects or in patients with a history of radiation therapy, such options are often not feasible. In these cases, free tissue transfer becomes the first choice (1).

Interestingly, the microsurgical reconstruction of the scalp was one of the earliest applications of free tissue transfer. Already in 1972, McLean and Buncke covered a large scalp defect with a free omentum majus flap (2). Microsurgery and its reconstructive possibilities have evolved since then, but some general principles still remain of unchanged importance.

Therefore, the aim of this review is to illustrate several general and specific considerations in patients undergoing microsurgical reconstruction of large oncological scalp defects.

## General Considerations

A sufficient preoperative planning is essential for an extensive surgical procedure, such as the oncological resection and microsurgical reconstruction of malignancies of the scalp.

First, the surgical resectability of the tumor needs to be evaluated. Although MRI provides good visualization of the soft tissues and the tumor, CT-scans can aid in assessing the amount of involvement of the calvarium bone. In patients with an extensive history of operative interventions, such as neck dissections or radiation therapy, an angiography of the possible recipient vessels can support preoperative decision-making and facilitate the intraoperative dissection. Based on the angiography findings (as well as in their absence), one should also devise a back-up plan in case the primary vessel can not be dissected due to scarring or does not show adequate flow after dissection.

Multiple soft-tissue and bone biopsies can aid in both an exact diagnosis of the malignancy and the planning of the surgical margins. Also, an adequate staging of the disease is paramount. Such complex cases should be preoperatively discussed in an interdisciplinary board of oncologists, neurosurgeons, and plastic surgeons. Here, not only the resection and reconstruction but also the use of (neo-)adjuvant radio- or chemotherapy should be discussed. Especially noteworthy in this regard is the trend toward less aggressive resections of sarcomas. Although the wide resection is still commonly recommended in sarcoma surgery, such an approach would render most of the sarcomas of the scalp to be considered not resectable. According to the findings in sarcomas of the extremities and recurrent sarcomas, less radical resections might be sufficient for most entities, as long as clear surgical margins are achieved (3, 4).

Only by incorporating all these findings, a reasonable treatment plan can be devised. However, even the most elaborate surgical treatment plan cannot stand on its own and needs to be adjusted to the individual patient and their respective wishes. Especially in a palliative situation, invasiveness of the procedure and its potential complications need to be weighted carefully against the expected gain in quality of life. Nonetheless, even large reconstructions should not be shunned in the face of palliation, if the patient is capable and willing to undertake such a journey. In this way, defects that are painful, bleeding, and demand extensive wound care can be covered, enabling the patient to regain their independence and quality of life (5, 6).

The possibility to cover nearly any oft-tissue defect of the scalp via free tissue transfer enables a radical surgical resection. Often, this resection also has to include the calvarium bone and the dura. Such defects are commonly covered by custom-made methyl acrylate implants or titanium mesh implants and dura patch plasties. In some cases, a microvascular transplant can be used as a dermal reinforcement of such a dura plasty (7).

## Soft-Tissue Reconstruction

**Figure 1:**
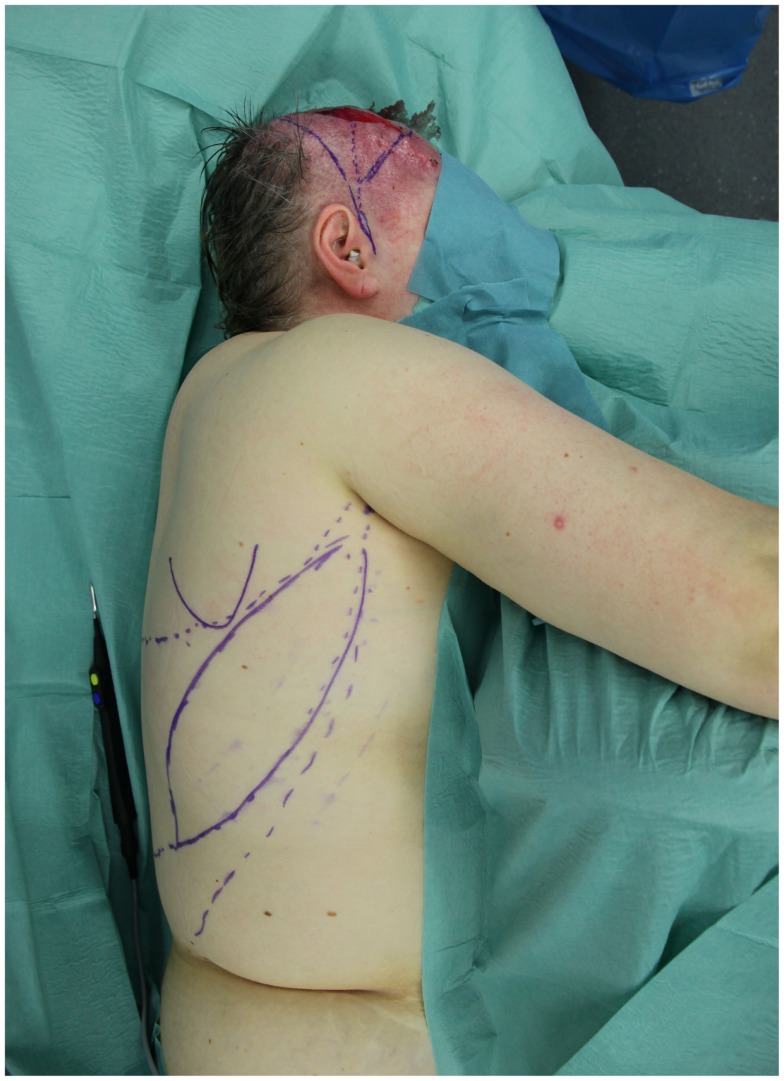
**Intraoperative view of a patient with an oncological defect of the scalp**. The latissimus skin paddle as well as the anatomical landmarks are marked.

**Figure 2:**
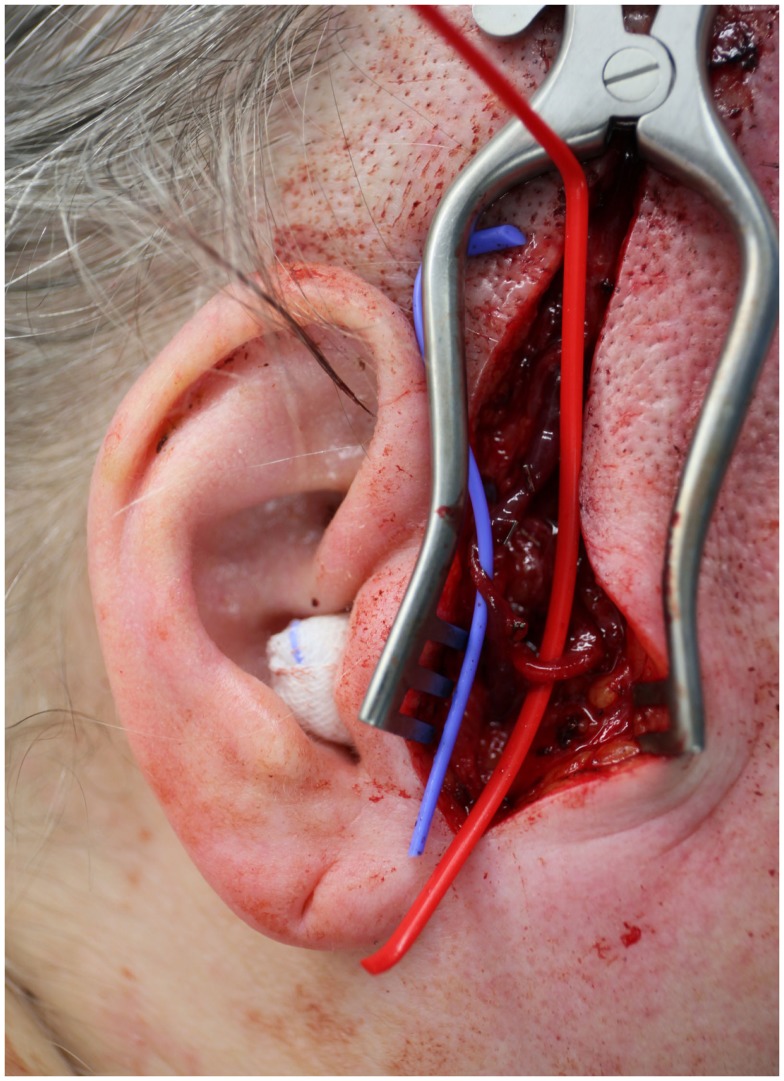
**The dissected superficial temporal vessels that will be utilized as recipient vessels**.

**Figure 3:**
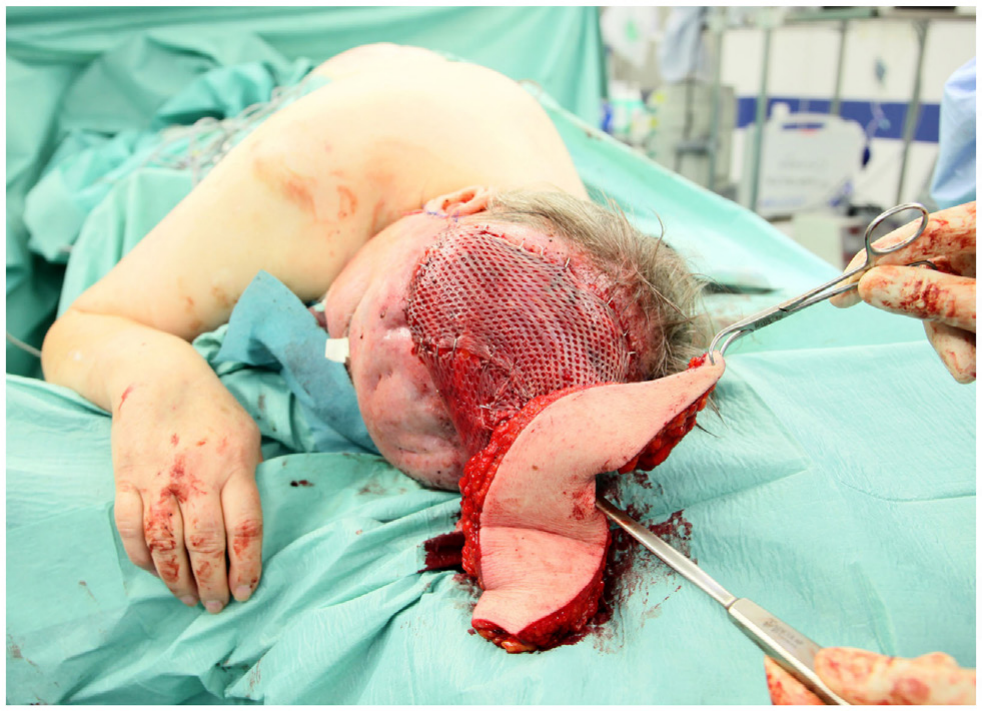
**After fitting of the flap**. Note the skin grafted muscle surface with leaves only small gaps for the vessels.

**Figure 4:**
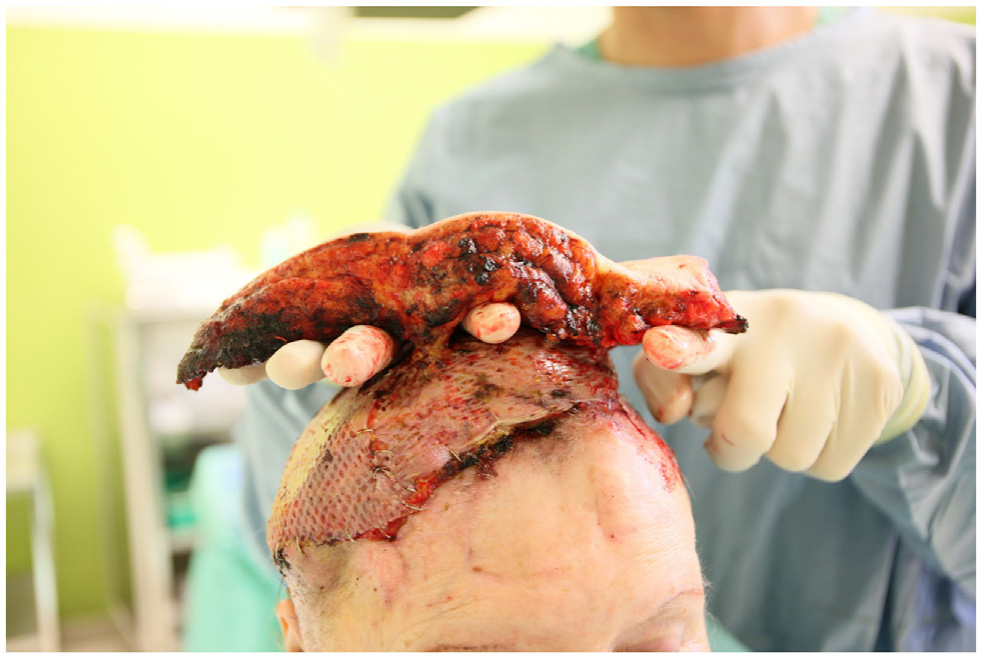
**Skin paddle of a latissimus dorsi free flap based on three perforator vessels 1 week after surgery**.

**Figure 5:**
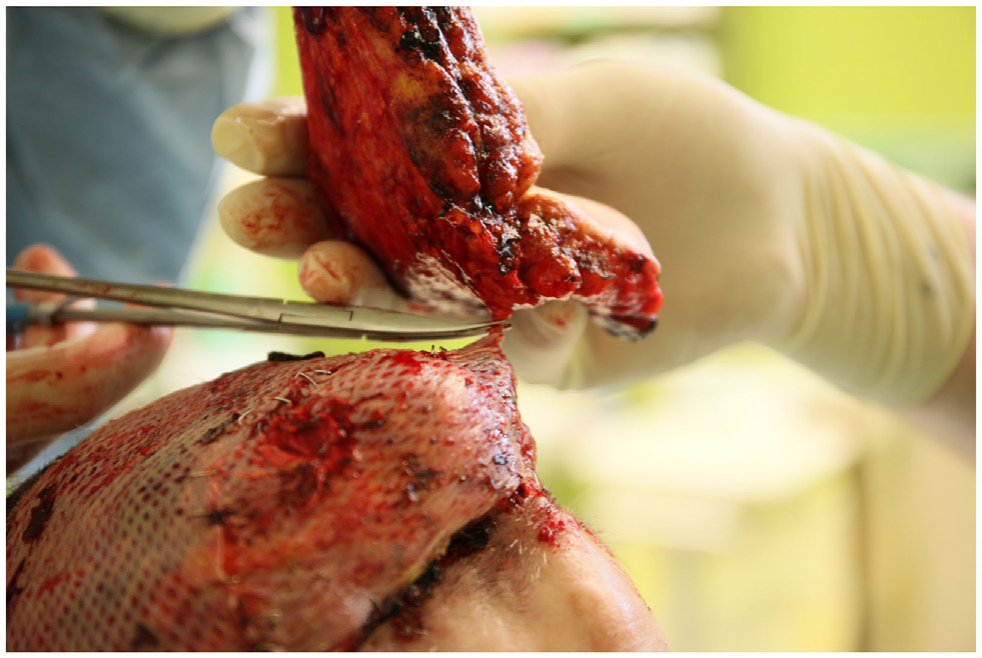
**Removal of the skin paddle after ligation and severing of the perforating vessels**.

A variety of free flaps have been described for the microsurgical reconstruction of the scalp (2, 8–10). The choice of free flap is mainly dependent on the size of the defect and the required pedicle length. Due to its size, the latissimus dorsi has become one of the work horse flaps for scalp reconstruction (11). It can be harvested as a sole muscle flap or musculocutaneous flap with an overlying skin paddle. Although this skin paddle facilitates clinical perfusion monitoring or/and could be use for extension of coverage, it is often very bulky due to the subcutaneous fat. In high risk cases requiring intense monitoring of the flap (coagulopathies, history of radiation, e.g.) such a skin paddle might be easier to monitor than the muscle itself. In such cases, one or more perforating vessel that supply the overlying skin paddle can be dissected in the initial operation. After isolating the paddle on these perforators, the muscle surface of the flap can be skin grafted, leaving only small gaps for the vessels (12). When the need for an intense clinical monitoring has subsided, the perforator-based skin paddle can be easily removed by ligating the vessels without the need for additional surgery. The initial bulk of the latissimus declines over the course of time and together with a split thickness graft results in a reasonable esthetic outcome. The case of a patient undergoing free latissimus dorsi coverage of the scalp following oncological resection is depicted in Figures 1–5.

In the beginning, we designed those skin islands on multiple perforators, but such large skin islands are often hard to fix to the underlying surface and can be compromised by shearing off due to their own weight. Therefore, a smaller island with one singled out perforator is often more feasible, especially since the option to use such a perforator island as another flap to close secondary defects is undesirable in this constellation due to the bulk of the subcutaneous fat.

**Figure 6:**
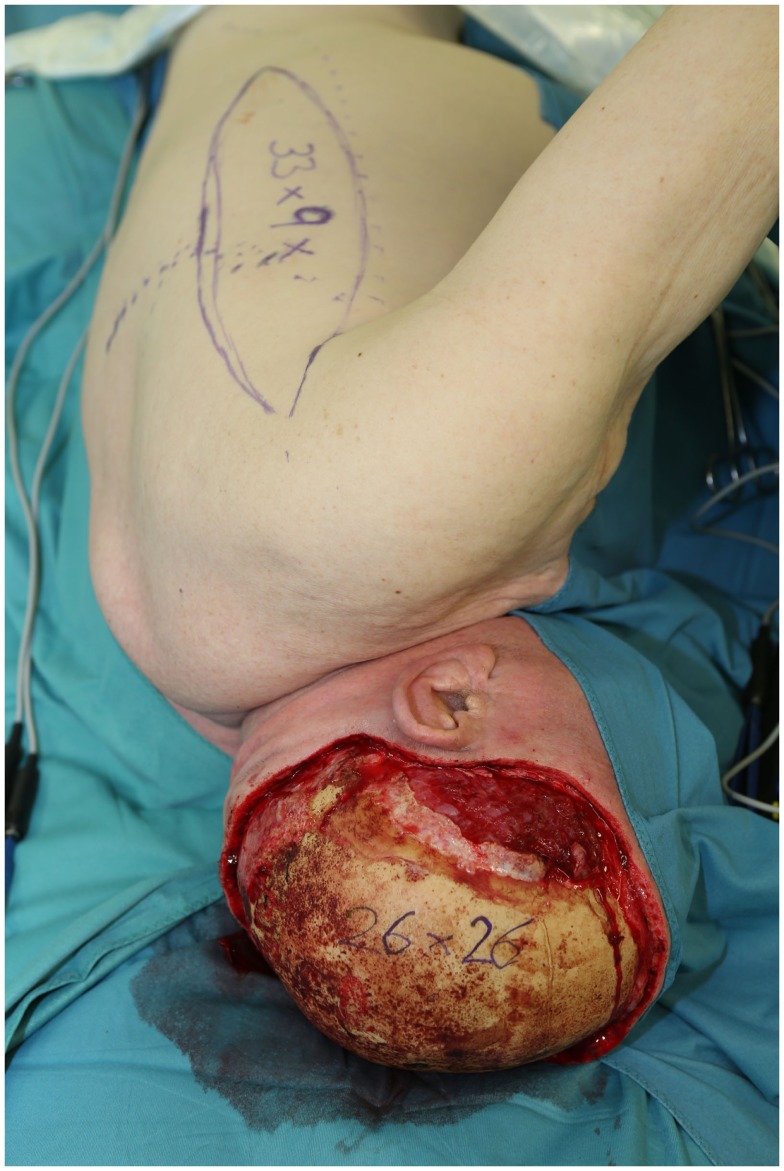
**Intraoperative view after total full thickness scalp resection due to an angiosarcoma**. Note the 26 cm × 26 cm sized defect and the planned parascapular flap with a size of 33 cm × 9 cm.

**Figure 7:**
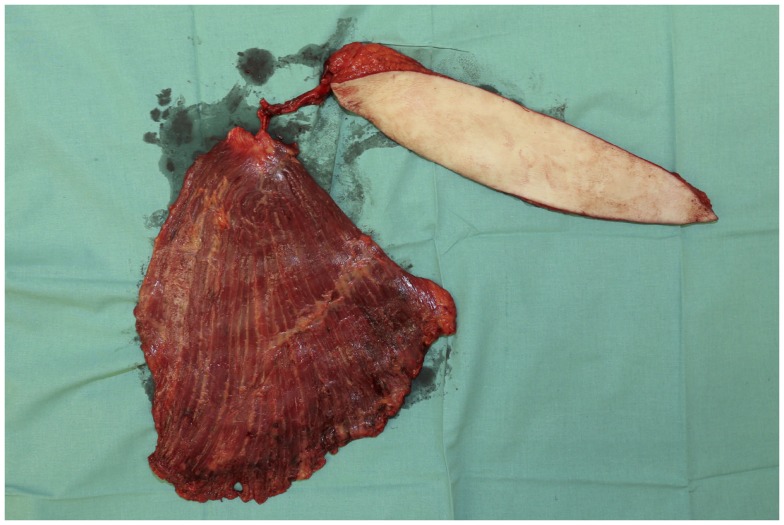
**The combined latissimus/parascapular flap after its elevation on the common vascular pedicle**.

**Figure 8:**
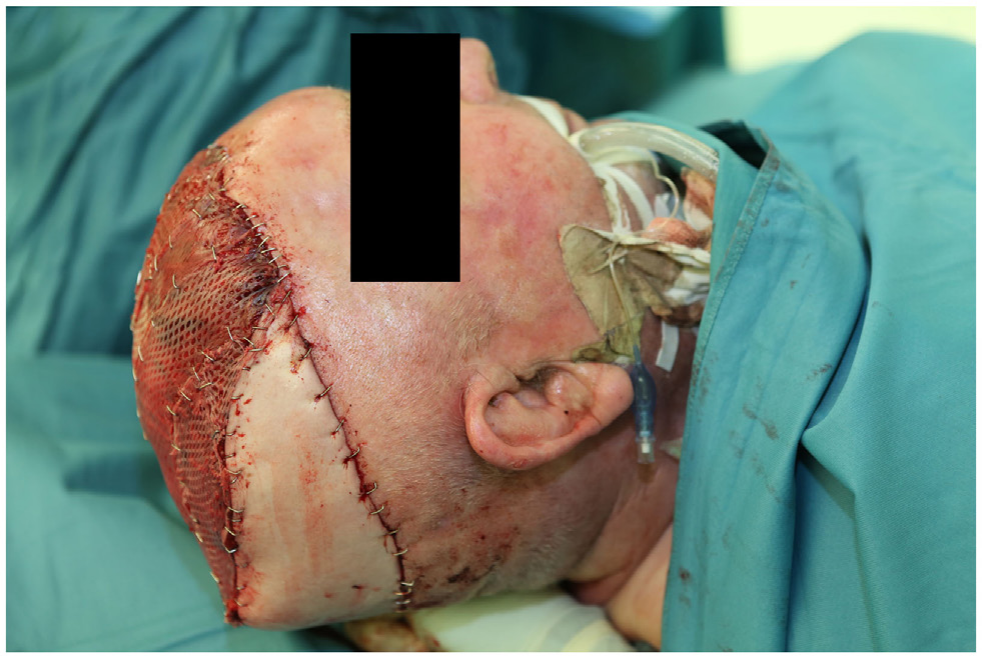
**Intraopearative view after fitting of the flaps**. Note the more resilient skin cover of the parascapular flap over the occiput on which the head rests.

**Figure 9:**
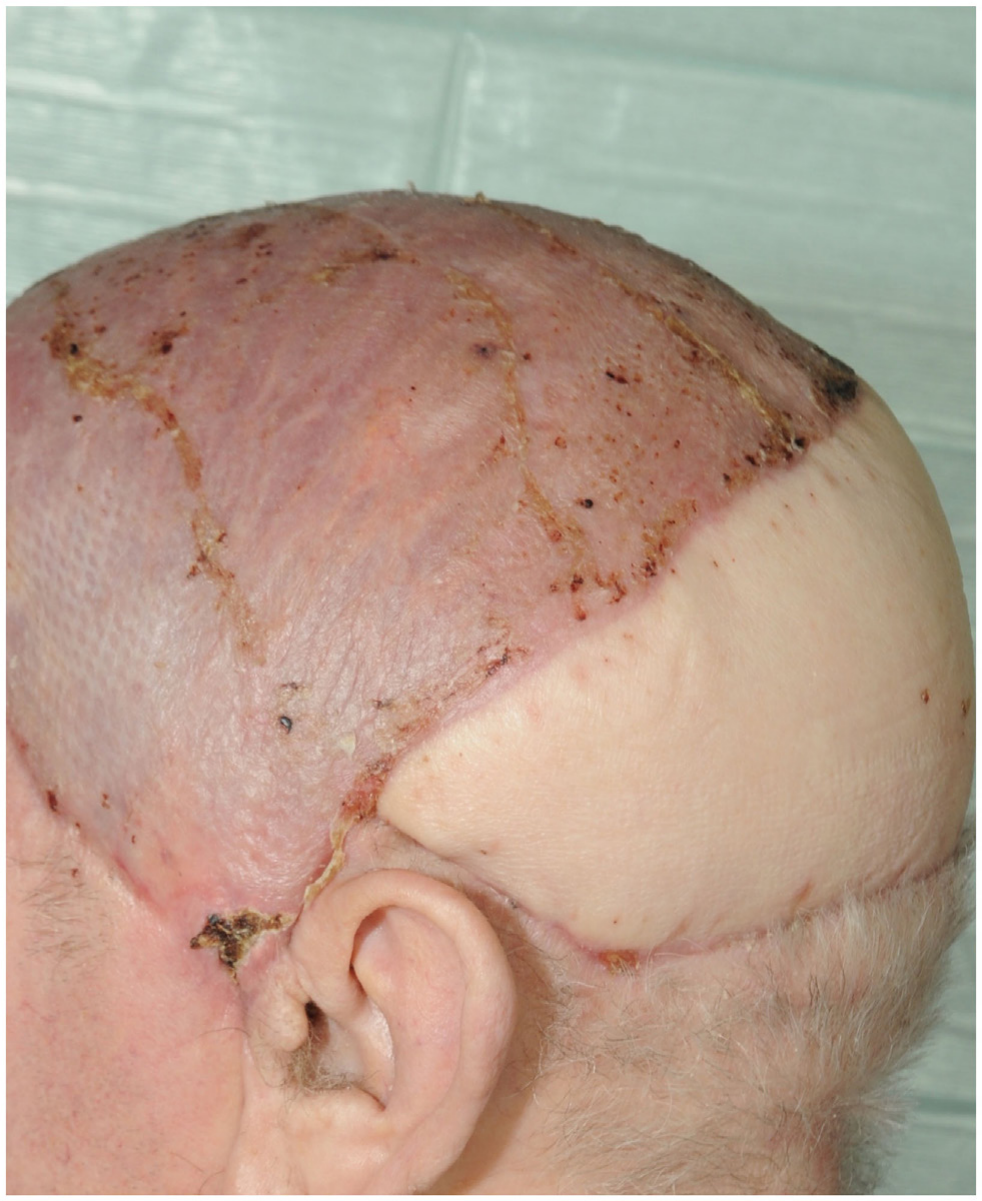
**Follow up after 4 weeks**.

Based on the same vascular tree, the (para-) scapular flap can be raised as a fascio-cutaneous flap that carries little bulk and sufficient pedicle length (13). Due to their common vascular tree, both the (para-)scapular and the latissimus flap can be harvested together, thus allowing for even larger defects to be covered. A disadvantage of harvesting the common vascular tree is the extensive diameter of the venular confluence and its mismatch with the tiny vein of the superficial temporal vessels. To achieve a smooth passage at the anastomosis, we utilize a chamfering technique that is discussed later. We prefer to place the parascapular flap with its good skin quality over the occipital bone and the latissimus muscle over the mid part of the calvarium. This way, the more resilient skin of the parascapular flap allows for a better cover when resting the head. Figures 6–9 depict the case of a patient undergoing total scalp resection for an angiosarcoma and the microsurgical reconstruction with a chimeric Latissimus/Parascapular flap.

Another commonly used free flap is the anterior-lateral thigh flap (ALT). The mean achievable flap size is smaller than in the latissimus; however, it carries less initial bulk in slender patients. In cases where obliteration of dead space is required, the flap can be raised as a musculocutaneous flap that incorporates the vastus lateralis muscle. One of the main advantages of the ALT flap over the latissimus and the (para-)scapular flap is the possibility to harvest it in a supine position. However, in extensive scalp defects, the patient is often positioned in either a prone or lateral position to allow access to the entire defect thus negating this advantage.

A good alternative for medium to large defects is the gracilis muscle free flap. It can be expanded and also flattened by intramuscular dissection, allowing for a stable coverage without too much bulk (14). Also, similar to the latissimus flap, the appearance of the grafted muscle surface is often superior to the patch-like appearance of fascio-cutaneous flaps.

Other potential flap choices include the ulnar and radial forearm flaps. Both deliver little bulk and often require a less complex dissection than perforator flaps. However, the donor site including the loss of a major artery can be problematic. Therefore, these flaps are not the first choice in our hands.

## Specific Considerations

One of the main things to be considered in microsurgical reconstructions is the recipient vessels. Although there are several potential vessels available in the head and neck, the choice of the optimal vessel depends on various factors. Depending on the localization of the defect, the recipient vessels should be easily reachable with the anticipated pedicle length, thus eliminating the need for vein grafts. In patients with extensive previous operations, such as neck dissections or history of radiotherapy, the integrity of the vessels might be compromised or its dissection can be hindered by scars. Therefore, preoperative evaluation of their patency is recommended in such cases. Often, hand-held Doppler assessment is sufficient. However, in selected cases, an angiography can aid in the preoperative planning. As mentioned above, sometimes the primary “go-to” vessel can not be dissected due to scarring or shows insufficient flow. Therefore, a back-up plan needs to be devised beforehand.

Due to its easy access in the preauricular space and reliable course, the superficial temporal artery is one of the most commonly used recipient vessels in microsurgical scalp reconstruction (10, 15). However, there can be a serious discrepancy of the caliber between the flap and the recipient vessels, especially in chimeric flaps. To reach an appropriate size of recipient vessels, one can either extend the dissection proximally, which in turn can bring the need for the interposition of vein grafts. Although such grafts carry an intrinsic risk, a proximal anastomosis often has less tendency for spasms and can deliver a higher blood flow to perfuse even very large flaps. Also, in our experience, the superficial temporal artery reacts quite sensitive to manipulation and its flow can easily become compromised due to vasoconstriction. However, by dissection of the artery to the level of the ear lobe the flow becomes sufficient in most cases.

**Figure 10:**
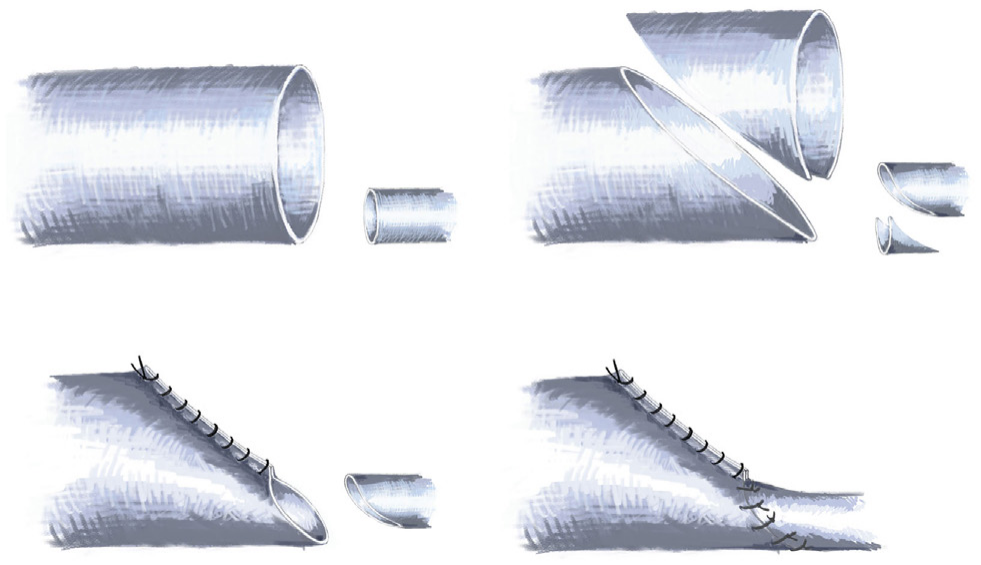
**Schematic drawing of the technique employed in cases of large caliber differences between recipient and flap vessels by chamfering the lumen**.

Another option is to perform an end-to-end anastomosis after preparing both vessels in the way depicted in Figure 10. By chamfering the larger lumen of the flap vessel, a cone-like configuration can be achieved. This cone can then be adapted to the size of the recipient vessels by the numbers of sutures placed. In this way, the lumen can be tailored to the required size in a safe and reliable way. Such a procedure does also carry some intrinsic risk for thrombosis, therefore careful consideration of the recipient vessels need to be taken.

Other options of recipient vessels include the superior thyroid and the facial artery. In cases where the concomitant veins are not sufficient, the internal and external jugular veins represent a reliable alternative (10, 16).

Due to the tendency to rest the head on the occipital bone, post-operative positioning of patients with total scalp reconstruction can be problematic. To avoid pressure on the flap, the patient can be positioned in a sitting position with some support of the cervical spine. If post-operative ventilation is required, the patient can be brought into a prone position to allow for an absolute pressure free environment for the flap. To facilitate this, special beds for the prone positioning of patients as seen in burn units can be used. However, both in sitting and prone positions, the risk for pressure sores is increased and therefore requires meticulous assessment of the soft tissues over high pressure areas.

**Figure 11:**
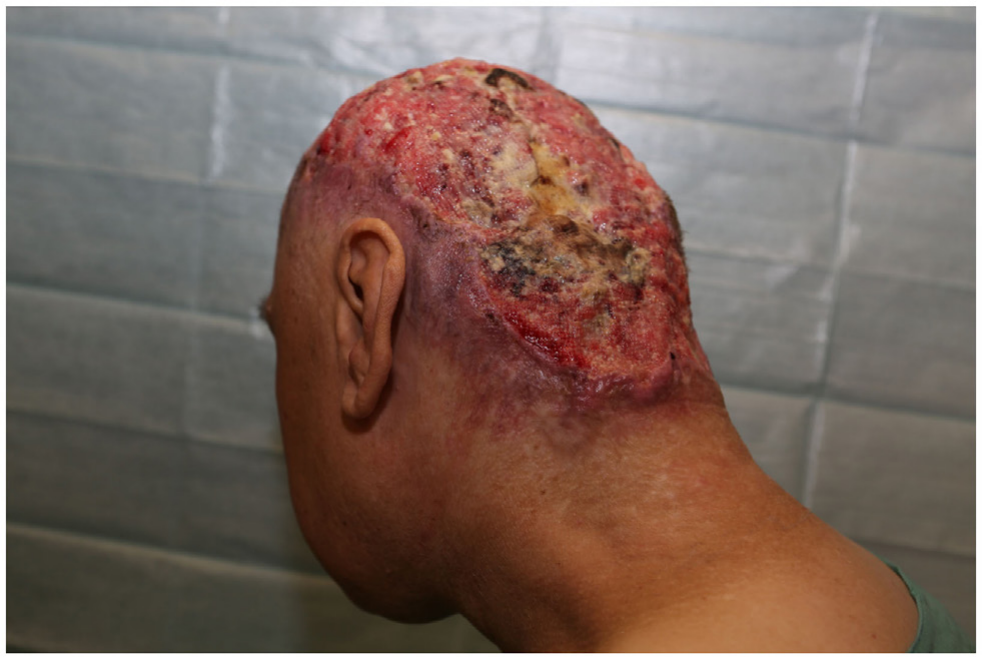
**A case of extensive squamous cell carcinoma of the scalp with infiltration of the calvarium**.

**Figure 12:**
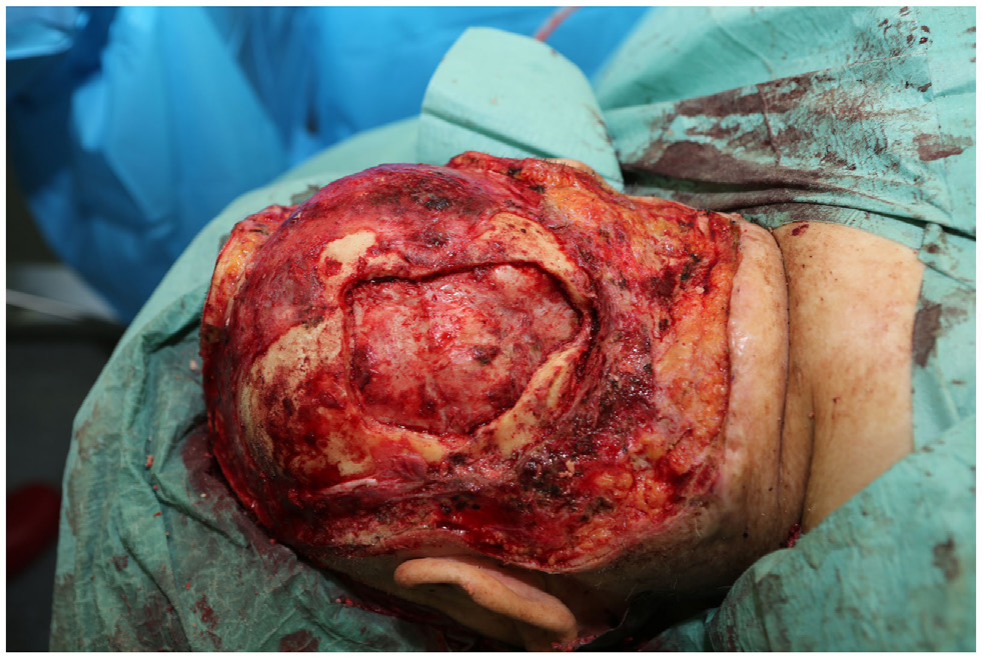
**Intraoperative view after resection of the scalp and the infiltrated calvarium**.

**Figure 13:**
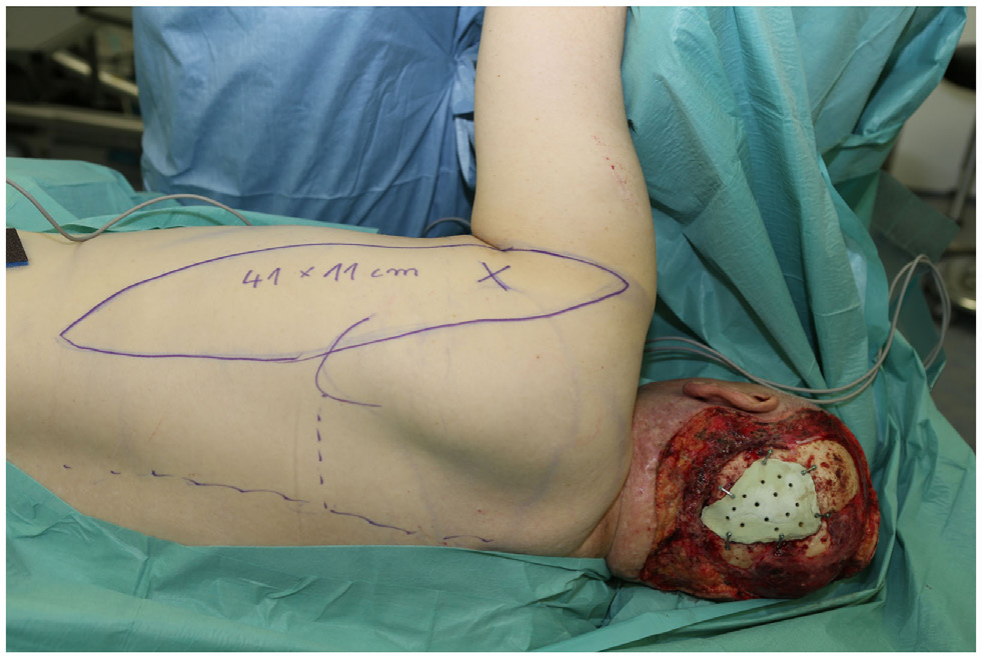
**The calvarial defect was closed with a custom-made methyl acrylate implant**. Note the anatomical landmarks and the outline of the planned skin paddle of the parascapular flap.

**Figure 14:**
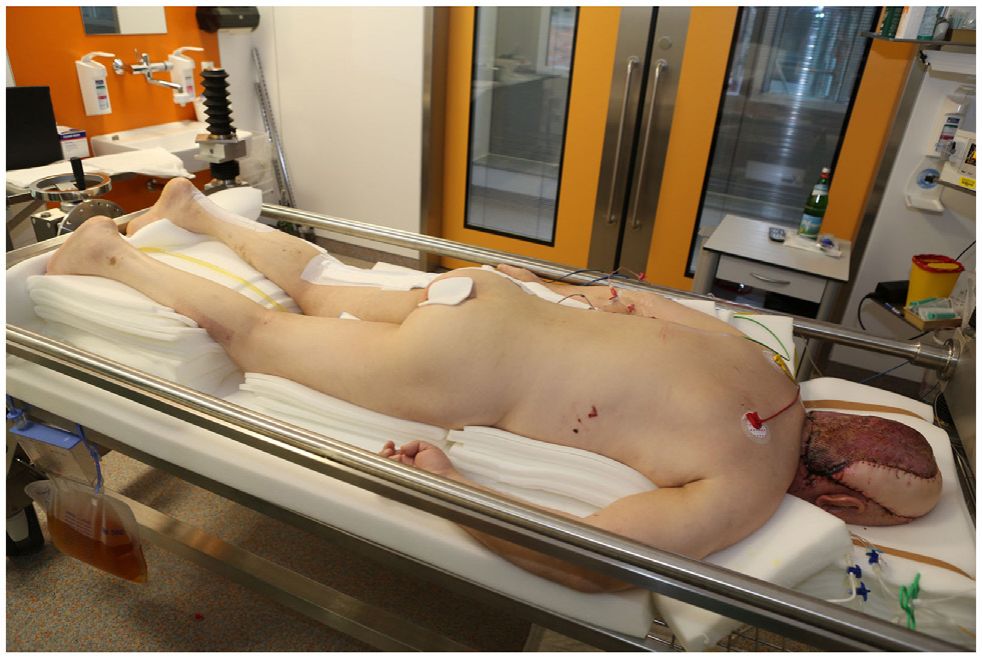
**The patient was initially positioned in a prone position**. Note the extensive padding to reduce the incidence of pressure sores and the apparatus on the end of the bed that facilitates rotating the bed.

**Figure 15:**
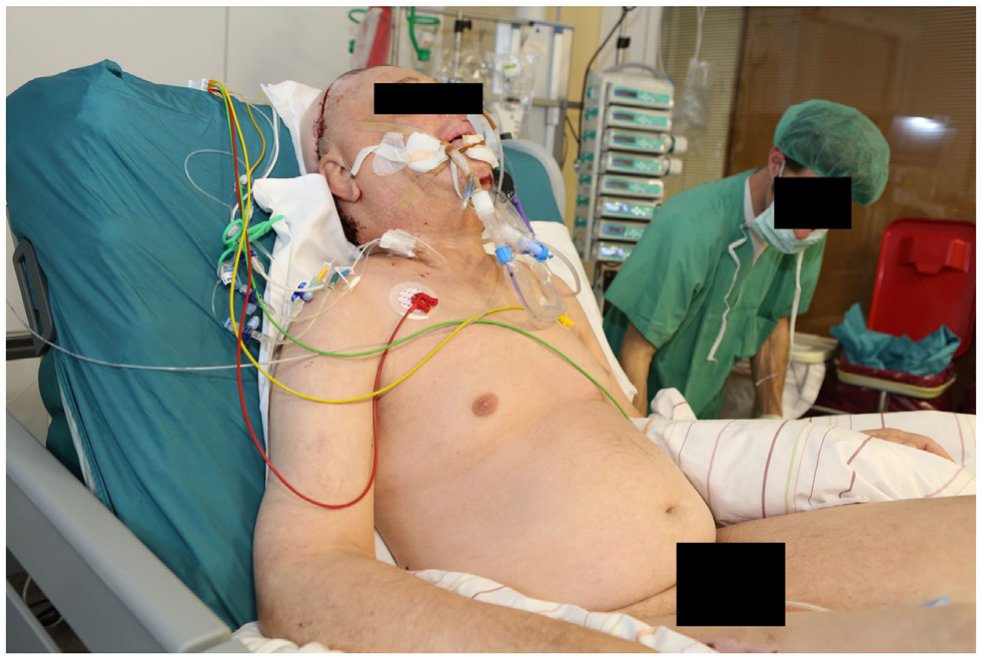
**The same patient in a sitting position to prepare for extubation**.

Figures 11–15 depict the case of a patient with a neglected carcinoma of the skull and a reduced compliance due to a mental handicap. Even despite complete resection of the malignancy, the patient deceased 12 months after surgery due to metastatic disease. However, the painful daily dressing changes were discontinued, improving the quality of life of the patient. Therefore, such complex reconstructions can also be feasible in a palliative setting. In such cases, the invasiveness of the procedure needs to be weighted very carefully against the expected gains in quality of life.

Also, the site of the anastomosis itself needs to be protected from any pressure. If the anastomosis is in the area of the lateral face and neck, a modified cervical spine collar with sufficient space over the area of the anastomosis can aid in protecting it.

## Conclusion

The ability to reconstruct even total scalp defects via free tissue transfer enables the radical surgical resection of malignancies.

A variety of free flaps are available for this task, most prominently the latissimus dorsi and the ALT flap. In very large defects, the latissimus dorsi and a parascapular flap can be elevated on their common vascular tree and used as a chimeric flap. Although the reconstruction itself as well as the perioperative management can be challenging, the overall outcomes with regards to the reconstruction is good.

## Conflict of Interest Statement

The authors declare that the research was conducted in the absence of any commercial or financial relationships that could be construed as a potential conflict of interest.
